# Sustainable data analysis with Snakemake

**DOI:** 10.12688/f1000research.29032.2

**Published:** 2021-04-19

**Authors:** Felix Mölder, Kim Philipp Jablonski, Brice Letcher, Michael B. Hall, Christopher H. Tomkins-Tinch, Vanessa Sochat, Jan Forster, Soohyun Lee, Sven O. Twardziok, Alexander Kanitz, Andreas Wilm, Manuel Holtgrewe, Sven Rahmann, Sven Nahnsen, Johannes Köster

**Affiliations:** 1 Algorithms for reproducible bioinformatics, Genome Informatics, Institute of Human Genetics, University Hospital Essen, University of Duisburg-Essen, Essen, Germany; 2 Institute of Pathology, University Hospital Essen, University of Duisburg-Essen, Essen, Germany; 3 Department of Biosystems Science and Engineering, ETH Zurich, Basel, Switzerland; 4 Swiss Institute of Bioinformatics (SIB), Basel, Switzerland; 5 EMBL-EBI, Hinxton, UK; 6 Broad Institute of MIT and Harvard, Cambridge, USA; 7 Department of Organismic and Evolutionary Biology, Harvard University, Cambridge, USA; 8 Stanford University Research Computing Center, Stanford University, Stanford, USA; 9 German Cancer Consortium (DKTK, partner site Essen) and German Cancer Research Center, DKFZ, Heidelberg, Germany; 10 Biomedical Informatics, Harvard Medical School, Harvard University, Boston, USA; 11 Charité - Universitätsmedizin Berlin, corporate member of Freie Universität Berlin, Humboldt-Universität zu Berlin, and Berlin Institute of Health (BIH), Center for Digital Health, Berlin, Germany; 12 Biozentrum, University of Basel, Basel, Switzerland; 13 SIB Swiss Institute of Bioinformatics / ELIXIR Switzerland, Lausanne, Switzerland; 14 Microsoft Singapore, Singapore, Singapore; 15 CUBI – Core Unit Bioinformatics, Berlin Institute of Health, Berlin, Germany; 16 Genome Informatics, Institute of Human Genetics, University Hospital Essen, University of Duisburg-Essen, Essen, Germany; 17 Quantitative Biology Center (QBiC), University of Tübingen, Tübingen, Germany; 18 Medical Oncology, Harvard Medical School, Harvard University, Boston, USA

**Keywords:** data analysis, workflow management, sustainability, reproducibility, transparency, adaptability, scalability

## Abstract

Data analysis often entails a multitude of heterogeneous steps, from the application of various command line tools to the usage of scripting languages like R or Python for the generation of plots and tables. It is widely recognized that data analyses should ideally be conducted in a reproducible way. Reproducibility enables technical validation and regeneration of results on the original or even new data. However, reproducibility alone is by no means sufficient to deliver an analysis that is of lasting impact (i.e., sustainable) for the field, or even just one research group. We postulate that it is equally important to ensure adaptability and transparency. The former describes the ability to modify the analysis to answer extended or slightly different research questions. The latter describes the ability to understand the analysis in order to judge whether it is not only technically, but methodologically valid.

Here, we analyze the properties needed for a data analysis to become reproducible, adaptable, and transparent. We show how the popular workflow management system Snakemake can be used to guarantee this, and how it enables an ergonomic, combined, unified representation of all steps involved in data analysis, ranging from raw data processing, to quality control and fine-grained, interactive exploration and plotting of final results.

## 1 Introduction

Despite the ubiquity of data analysis across scientific disciplines, it is a challenge to ensure *in silico* reproducibility ^1– 3^. By automating the analysis process, workflow management systems can help to achieve such reproducibility. Consequently, a “Cambrian explosion” of diverse scientific workflow management systems is in process; some are already in use by many and evolving, and countless others are emerging and being published (see https://github.com/pditommaso/awesome-pipeline). Existing systems can be partitioned into five niches which we will describe below, with highlighted examples of each.

First, workflow management systems like Galaxy ^4^, KNIME ^5^, and Watchdog ^6^ offer graphical user interfaces for composition and execution of workflows. The obvious advantage is the shallow learning curve, making such systems accessible for everybody, without the need for programming skills.

Second, with systems like Anduril ^7^, Balsam ^8^, Hyperloom ^9^, Jug ^10^, Pwrake ^11^, Ruffus ^12^, SciPipe ^13^, SCOOP ^14^, and COMPSs ^15^, and JUDI ^16^, workflows are specified using a set of classes and functions for generic programming languages like Python, Scala, and others. Such systems have the advantage that they can be used without a graphical interface (e.g. in a server environment), and that workflows can be straightforwardly managed with version control systems like Git ( https://git-scm.com).

Third, with systems like Nextflow ^17^, Snakemake ^18^, BioQueue ^19^, Bpipe ^20^, ClusterFlow ^21^, Cylc ^22^, and BigDataScript ^23^, workflows are specified using a domain specific language (DSL). Here, the advantages of the second niche are shared, while adding the additional benefit of improved readability; the DSL provides statements and declarations that specifically model central components of workflow management, thereby obviating superfluous operators or boilerplate code. For Nextflow and Snakemake, since the DSL is implemented as an extension to a generic programming language (Groovy and Python), access to the full power of the underlying programming language is maintained (e.g. for implementing conditional execution and handling configuration).

Fourth, with systems like Popper ^24^, workflow specification happens in a purely declarative way, via configuration file formats like YAML ^25^. These declarative systems share the concision and clarity of the third niche. In addition, workflow specification can be particularly readable for non-developers. The caveat of these benefits is that by disallowing imperative or functional programming, these workflow systems can be more restrictive in the processes that can expressed.

Fifth, there are system-independent workflow specification languages like CWL ^26^ and WDL ^27^. These define a declarative syntax for specifying workflows, which can be parsed and executed by arbitrary executors, e.g. Cromwell ( https://cromwell.readthedocs.io), Toil ^28^, and Tibanna ^29^. Similar to the fourth niche, a downside is that imperative or functional programming is not or less integrated into the specification language, thereby limiting the expressive power. In contrast, a main advantage is that the same workflow definition can be executed on various specialized execution backends, thereby promising scalability to virtually any computing platform. Another important use case for system-independent languages is that they promote interoperability between other workflow definition languages. For example, Snakemake workflows can (within limits) be automatically exported to CWL, and Snakemake can make use of CWL tool definitions. An automatic translation of any CWL workflow definition into a Snakemake workflow is planned as well.

Today, several of the above mentioned approaches support full *in silico* reproducibility of data analyses (e.g. Galaxy, Nextflow, Snakemake, WDL, CWL), by allowing the definition and scalable execution of each involved step, including deployment of the software stack needed for each step (e.g. via the Conda package manager, https://docs.conda.io, Docker, https://www.docker.com, or Singularity ^30^ containers).

**Figure 1.**
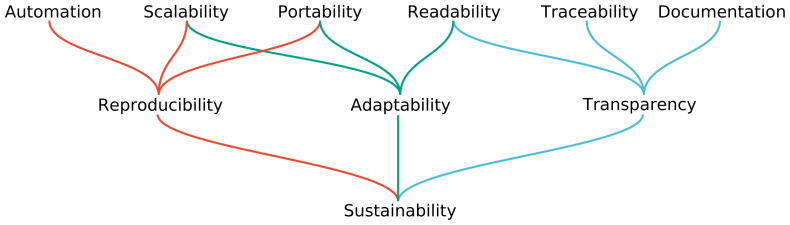
Hierarchy of aspects to consider for sustainable data analysis. By supporting the top layer, a workflow management system can promote the center layer, and thereby help to obtain true sustainability.

Reproducibility is important to generate trust in scientific results. However, we argue that a data analysis is only of lasting and sustained value for the authors and the scientific field if a hierarchy of additional interdependent properties is ensured ( Figure 1).

First, to gain full *in silico reproducibility*, a data analysis has to be *automated*, *scalable* to various computational platforms and levels of parallelism, and *portable* in the sense that it is able to be automatically deployed with all required software in exactly the needed versions.

Second, while being able to reproduce results is a major achievement, *transparency* is equally important: the validity of results can only be fully assessed if the parameters, software, and custom code of each analysis step are fully accessible. On the level of the code, a data analysis therefore has to be *readable* and well- *documented*. On the level of the results it must be possible to *trace* parameters, code, and components of the software stack through all involved steps.

Finally, valid results yielded from a reproducible data analysis have greater meaning to the scientific community if the analysis can be reused for other projects. In practice, this will almost never be a plain reuse, and instead requires *adaptability* to new circumstances, for example, being able to extend the analysis, replace or modify steps, and adjust parameter choices. Such adaptability can only be achieved if the data analysis can easily be executed in a different computational environment (e.g. at a different institute or cloud environment), thus it has to be *scalable* and *portable* again (see Figure 1). In addition, it is crucial that the analysis code is as *readable* as possible such that it can be easily modified.

**Figure 2.**
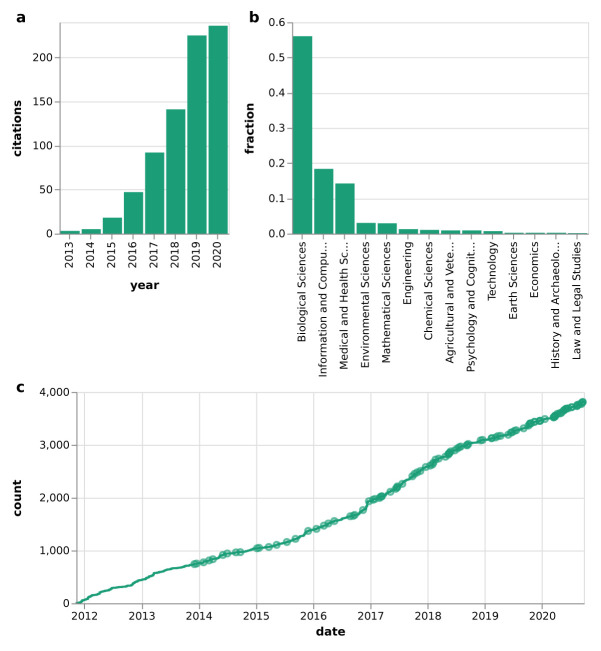
Citations and development of Snakemake. ( **a**) citations by year of the original Snakemake article (note that the year 2020 is still incomplete at the time of writing). ( **b**) citations by scientific discipline of the citing article. Data source: https://badge.dimensions.ai/details/id/pub.1018944052, 2020/09/29. ( **c**) cumulative number of git commits over time; Releases are marked as circles.

In this work, we show how data analysis sustainability in terms of these aspects is supported by the open source workflow management system Snakemake ( https://snakemake.github.io). Since its original publication in 2012, Snakemake has seen hundreds of releases and contributions ( Figure 2c). It has gained wide adoption in the scientific community, culminating in, on average, more than five new citations per week, and over 700 citations in total ( Figure 2a,b). This makes Snakemake one of the most widely used workflow management systems in science.

In order to address the requirements of a potentially diverse readership, we decided to split the following content into two parts. Section 2 concisely presents Snakemake in terms of the aspects shown in Figure 1, whereas section 3 provides further details for the particularly interested reader, including how to compose multiple workflows for integrative data analysis, advanced design patterns, and additional technical details.

## 2 Methods and results

We present how Snakemake enables the researcher to conduct data analyses that have all the properties leading to reproducibility, transparency and adaptability. This in turn allows the analysis to become a sustainable resource both for the researcher themselves and the scientific community. We structure the results by each of the properties leading to sustainable data analyses ( Figure 1).

 We will thereby introduce relevant features of both the workflow definition language as well as the execution environment. Several of them are shared with other tools, while others are (at the time of writing) exclusive to Snakemake. Finally, there are features that other workflow management systems provide while Snakemake does not (or not yet) offer them. We intentionally refrain from performing a full comparison with other tools, as we believe that such a view will never be unbiased (and quickly outdated), and should instead be provided by review articles or performed by the potential users based on their individual needs.

### 2.1 Automation

**Figure 3.**
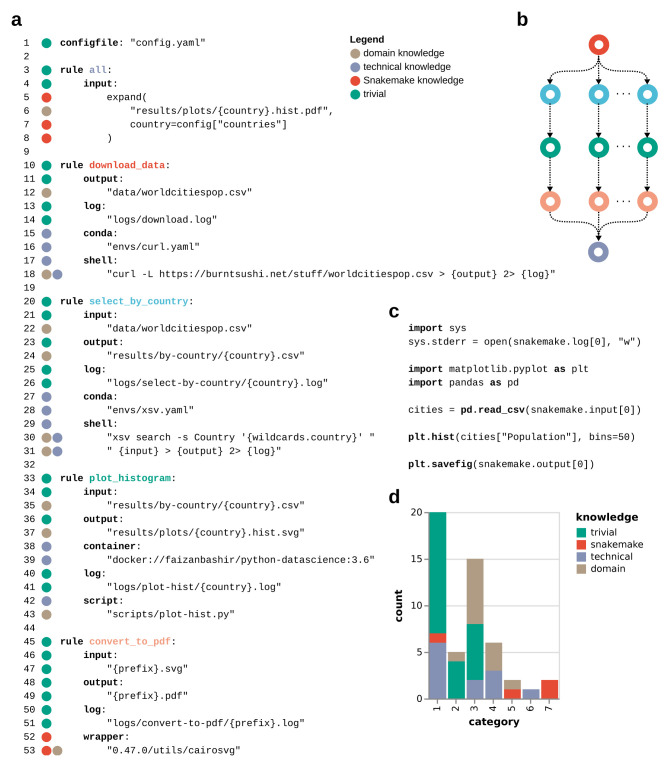
Example Snakemake workflow. ( **a**) workflow definition; hypothesized knowledge requirement for line readability is color-coded on the left next to the line numbers. ( **b**) directed acyclic graph (DAG) of jobs, representing the automatically derived execution plan from the example workflow; job node colors reflect rule colors in the workflow definition. ( **c**) content of script plot-hist.py referred from rule plot_histogram. ( **d**) knowledge requirements for readability by statement category (see subsection 3.3). The example workflow downloads data, plots histograms of city populations within a given list of countries, and converts these from SVG to PDF format. Note that this is solely meant as a short yet comprehensive demonstration of the Snakemake syntax.

The central idea of Snakemake is that workflows are specified through decomposition into steps represented as *rules* ( Figure 3). Each rule describes how to obtain a set of output files from a set of input files. This can happen via a shell command, a block of Python code, an external script (Python, R, or Julia), a Jupyter notebook ( https://jupyter.org), or a so-called wrapper (see Sec. 2.2.1). Depending on the computing platform used and how Snakemake is configured, input and output files are either stored on disk, or in a remote storage (e.g. FTP, Amazon S3, Google Storage, Microsoft Azure Blob Storage, etc.). Through the use of wildcards, rules can be generic. For example, see the rule select_by_country in Figure 3a (line 20). It can be applied to generate any output file of the form results/by-country/{country}.csv, with {country} being a wildcard that can be replaced with any non-empty string. In shell commands, input and output files, as well as additional parameters, are directly accessible by enclosing the respective keywords in curly braces (in case of more than a single item in any of these, access can happen by name or index).

When using script integration instead of shell commands, Snakemake automatically inserts an object giving access to all properties of the job (e.g. snakemake.output[0], see Figure 3c). This avoids the presence and repetition of boiler plate code for parsing command line arguments. By replacing wildcards with concrete values, Snakemake turns any rule into a job which will be executed in order to generate the defined output files.

Dependencies between jobs are implicit, and inferred automatically in the following way. For each input file of a job, Snakemake determines a rule that can generate it— for example by replacing wildcards again (ambiguity can be resolved by prioritization or constraining wildcards)— yielding another job. Then, Snakemake goes on recursively for the latter, until all input files of all jobs are either generated by another job or already present in the used storage (e.g., on disk). Where necessary, it is possible to provide arbitrary Python code to infer input files based on wildcard values or even the contents of output files generated by upstream jobs.

From this inference, Snakemake obtains a directed acyclic graph of jobs (DAG, see Figure 3b). The time needed for this is linear in the number of jobs involved in the workflow, and negligible compared to the usual runtimes of the workflow steps (see subsection 3.5).

Figure 3a illustrates all major design patterns needed to define workflows with Snakemake: workflow configuration (line 1), aggregations (line 5–8), specific (line 33–43) and generic (line 45–53) transformations, target rules (line 3–8), log file definition, software stack definition, as well as shell command, script, and wrapper integration. subsection 3.2 presents additional patterns that are helpful in certain situations (e.g. conditional execution, iteration, exploration of large parameter spaces, benchmarking, scatter/gather).

***2.1.1 Automated unit test generation.*** When maintaining and developing a production workflow, it is important to test each contained step, ideally upon every change to the workflow code. In software development, such tests are called *unit tests* ^31^. From a given source workflow with already computed results that have been checked for correctness, Snakemake can automatically generate a suite of unit tests, which can be executed via the Pytest framework ( https://pytest.org). Each unit test consists of the execution of one rule, using input data taken from the source workflow. The generated results are by default compared byte-by-byte against the results given by in the source workflow. However, this behavior can be overwritten by the user. It is advisable to keep the input datasets of the source workflow small in order to ensure that unit tests finish quickly.

### 2.2 Readability

The workflow definition language of Snakemake is designed to allow maximum readability, which is crucial for transparency and adaptability. For natural-language readability, the occurrence of known words is important. For example, the Dale-Chall readability formula derives a score from the fraction of potentially unknown words (that do not occur in a list of common words) among all words in a text ^32^. For workflow definition languages, one has to additionally consider whether punctuation and operator usage is intuitively understandable. When analyzing the above example workflow ( Figure 3a) under these aspects, code statements fall into seven categories ( subsection 3.3). In addition, for each statement, we can judge whether it

1. needs domain knowledge (from the field analyzed in the given workflow),
2. needs technical knowledge (e.g. about Unix-style shell commands or Python),
3. needs Snakemake knowledge,
4. is trivial (i.e., it should be understandable for everybody).

In Figure 3, we hypothesize the required knowledge for readability of each code line. Most statements are understandable with either general education, domain, or technical knowledge. In particular, only six lines need Snakemake-specific knowledge ( Figure 3d). The rationale for each hypothesis can be found in subsection 3.3.

 It should be highlighted that with production workflows, there will always be parts of the codebase that go beyond the simple example shown here, for example by using advanced design patterns ( subsection 3.2), or various Python functions for retrieving parameter values, per-sample configurations, etc. Since Snakemake supports modularization of workflow definitions ( subsubsection 2.2.1), it is however possible to hide more technical parts of the workflow definition (e.g. helper functions or variables) from readers that are just interested in a general overview. This way, workflows can try to keep a ratio between the different types of knowledge requirements that is similar to this example, while still allowing to easily enter the more complicated parts of the codebase. In the shown example, a good candidate for such a strategy is the lambda expression ( Figure 3a, line 39) for retrieving the number of bins per country from the workflow configuration. While the used way of definition requires specific knowledge about Snakemake (and Python) when trying to understand the line, it can be simplified for a reader that just wants to get an overview of the workflow by replacing the statement with a function name, for example get_bins and moving the actual function into a separate file which is included into the main workflow definition (see subsubsection 2.2.1).

Since dependencies between jobs are implicitly encoded via matching filename patterns, we hypothesize that, in many cases, no specific technical knowledge is necessary to understand the connections between the rules. The file-centric description of workflows makes it intuitive to to infer dependencies between steps; when the input of one rule reoccurs as the output of another, their link and order of execution is clear. Again, one should note that this holds for simple cases as in this example. Conditional dependencies, input functions, etc. (see subsection 3.2), can easily yield dependencies that are more complex to understand. Also, such a textual definition does not immediately show the entire dependency structure of a workflow. It is rather suited to zoom in on certain steps, e.g., for understanding or modifying them. Therefore, Snakemake provides mechanisms that help with understanding dependencies on a global level (e.g. allowing to visualize them via the command line, or by automatically generating interactive reports).

In summary, the readability of the example in Figure 3 should be seen as an optimum a Snakemake workflow developer should aim for. Where the optimum cannot be reached, modularization should be used to help the reader to focus on parts that are understandable for her or his knowledge and experience. Further, such difficulties can be diminished by Snakemake’s ability to automatically generate interactive reports that combine codebase and results in a visual way ( subsection 2.4) and thereby help to explore specific parts of the codebase (e.g. to look up the code used for generating a particular plot) and dependencies without the need to understand the entire workflow.

***2.2.1 Modularization***. In practice, data analysis workflows are usually composed of different parts that can vary in terms of their readability for different audiences. Snakemake offers various levels of modularization that help to design a workflow in a way that ensures that a reader is not distracted from the aspects relevant for her or his interest.

**Snakefile inclusion.** A Snakemake workflow definition (a so-called Snakefile) can include other Snakefiles via a simple include statement that defines their path or URL. Such inclusion is best used to separate a workflow into sets of rules that handle a particular part of the analysis by working together. By giving the included Snakefiles speaking names, they enable the reader to easily navigate to the part of the workflow she or he is interested in.

**Workflow composition.** By declaring so-called workflow modules, Snakemake allows to compose multiple external workflows together, while modifying and extending them on the fly and documenting those changes transparently. A detailed description of this mechanism can be found in subsection 3.1.

**Step-wise modularization.** Some workflow steps can be quite specific and unique to the analysis. Others can be common to the scientific field and utilize widely used tools or libraries in a relatively standard way. For the latter, Snakemake provides the ability to deposit and use *tool wrappers* in/from a central repository. In contrast, the former can require custom code, often written in scripting languages like R or Python. Snakemake allows the user to modularize such steps either into scripts or to craft them interactively by integrating with Jupyter notebooks ( https://jupyter.org). In the following, we elaborate on each of the available mechanisms.

**Script integration.** Integrating a script works via a special script directive (see Figure 3a, line 42). The referred script does not need any boilerplate code, and can instead directly use all properties of the job (input files, output files, wildcard values, parameters, etc.), which are automatically inserted as a global snakemake object before the script is executed (see Figure 3c).

**Jupyter notebook integration.** Analogous to script integration, a notebook directive allows a rule to specify a path to a Jupyter notebook. Via the command line interface, it is possible to instruct Snakemake to open a Jupyter notebook server for editing a notebook in the context of a specific job derived from the rule that refers to the notebook. The notebook server can be accessed via a web browser in order to interactively program the notebook until the desired results (e.g. a certain plot or figure) are created as intended. Upon saving the notebook, Snakemake generalizes it such that other jobs from the same rule can subsequently re-use it automatically without the need for another interactive notebook session.

**Tool wrappers.** Reoccurring tools or libraries can be shared between workflows via Snakemake tool wrappers (see Figure 3a, line 52–53). A central public repository ( https://snakemake-wrappers.readthedocs.io) allows the community to share wrappers with each other. Each wrapper consists of a Python or R script that either uses libraries of the respective scripting language or calls a shell command. Moreover, each wrapper provides a Conda environment defining the required software stack, including tool and library versions (see subsection 2.3). Often, shell command wrappers contain some additional code that works around various idiosyncrasies of the wrapped tool (e.g. dealing with temporary directories or converting job properties into command line arguments). A wrapper can be used by simply copying and adapting a provided example rule (e.g. by modifying input and output file paths). Upon execution, the wrapper code and the Conda environment are downloaded from the repository and automatically deployed to the running system. In addition to single wrappers, the wrapper repository also offers pre-defined, tested combinations of wrappers that constitute entire sub-workflows for common tasks (called meta-wrappers). This is particularly useful for combinations of steps that reoccur in many data analyses. All wrappers are automatically tested to run without errors prior to inclusion in the repository, and upon each committed change.

***2.2.2 Standardized code linting and formatting.*** The readability of programming code can be heavily influenced by adhering to common style and best practices ^33^. Snakemake provides automatic code formatting (via the tool snakefmt) of workflows, together with any contained Python code. Snakefmt formats plain Python parts of the codebase with the Python code formatter Black ( https://black.readthedocs.io), while providing its own formatting for any Snakemake specific syntax. Thereby, Snakefmt aims to ensure good readability by using one line per input/output file or parameter, separating global statements like rules, configfiles, functions etc. with two empty lines (such that they appear as separate blocks), and breaking too long lines into shorter multi-line statements.

In addition, Snakemake has a built in *code linter* that detects code violating best practices and provides suggestions on how to improve the code. For example, this covers missing directives (e.g. no software stack definition or a missing log file), indentation issues, missing environment variables, unnecessarily complicated Python code (e.g. string concatenations), etc. 

Both formatting and linting should ideally be checked for in continuous integration setups, for example via Github Actions ( https://github.com/features/actions). As such, there are preconfigured Github actions available for both Snakefmt ( https://github.com/snakemake/snakefmt#github-actions) and the code linter ( https://github.com/snakemake/snakemakegithub-action#example-usage). 

### 2.3 Portability

Being able to deploy a data analysis workflow to an unprepared system depends on: (a) the ability to install the workflow management system itself, and (b) the ability to obtain and use the required software stack for each analysis step. Snakemake itself is easily deployable via the Conda package manager ( https://conda.io), as a Python package ( https://pypi.io), or a Docker container ( https://hub.docker.com/r/snakemake/snakemake). Instructions and further information can be found at https://snakemake.github.io. 

The management of software stacks needed for individual rules is directly integrated into Snakemake itself, via two complementary mechanisms.

**Conda integration** For each rule, it is possible to define a software environment that will be automatically deployed via the Conda package manager (via a conda directive, see Figure 3a, line 15). Each environment is described by a lightweight YAML file used by conda to install constituent software. While efficiently sharing base libraries like Glib with the underlying operating system, software defined in the environment takes precedence over the same software in the operating system, and is isolated and independent from the same software in other Conda environments.

**Container integration** Instead of defining Conda environments, it is also possible to define a container for each rule (via a container directive, see Figure 3a, line 38). Upon execution, Snakemake will pull the requested container image and run a job inside that container using Singularity ^30^. The advantage of using containers is that the execution environment can be controlled down to the system libraries, and becomes portable across operating systems, thereby further increasing reproducibility ^34^. Containers already exist in centralized repositories for a wide range of scientific software applications, allowing easy integration info Snakemake workflows.

**Automatic containerization** The downside of using containers is that generating and modifying container images requires additional effort, as well as storage, since the image has to be uploaded to a container registry. Moreover, containers limit the adaptability of a pipeline, since it is less straightforward and ad hoc to modify them. Therefore, we advice to rely on Conda during the development of a workflow, while required software environments may rapidly evolve. Once a workflow becomes production ready or is published, Snakemake offers the ability to automatically generate a containerized version. For this, Snakemake generates a Dockerfile that deploys all defined Conda environments into the container. Once the corresponding container image has been built and uploaded to a container registry, it can be used in the workflow definition via the containerized directive. Upon workflow execution, Snakemake will then use the Conda environments that are found in the container, instead of having to recreate them.

### 2.4 Traceability and documentation

While processing a workflow, Snakemake tracks input files, output files, parameters, software, and code of each executed job. After completion, this information can be made available via self-contained, interactive, HTML based reports. Output files in the workflow can be annotated for automatic inclusion in the report. These features enable the interactive exploration of results alongside information about their provenance. Since results are included into the report, their presentation does not depend on availability of server backends, making Snakemake reports easily portable and archivable.

First, the report enables the interactive exploration of the entire workflow, by visualizing the dependencies between rules as a graph. Thereby, the nodes of the graph can be clicked in order to show details about the corresponding rules, like input and output files, software stack (used container image or conda environment), and shell, script, or notebook code. Second, the reports shows runtime statistics for all executed jobs. Third, used configuration files can be viewed. Fourth, the report shows the included output files (e.g. plots and tables), along with job specific information (rule, wildcard values, parameters), previews of images, download functionality, and a textual description. The latter can be written via a templating mechanism (based on Jinja2, https://jinja.palletsprojects.com) which allows to dynamically react on wildcard values, parameters and workflow configuration.

An example report summarizing the data analysis conducted for this article can be found at https://doi.org/10.5281/zenodo.4244143 ^35^. In the future, Snakemake reports will be extended to additionally follow the RO-crate standard, which will make them machine-readable and allow an integration with web services like https://workflowhub.eu. 

### 2.5 Scalability

Being able to scale a workflow to available computational resources is crucial for reproducing previous results as well as adapting a data analysis to novel research questions or datasets. Like many other state-of-the-art workflow management systems, Snakemake allows workflow execution to scale to various computational platforms (but not to combine multiple of them in a single run), ranging from single workstations to large compute servers, any common cluster middleware (like Slurm, PBS, etc.), grid computing, and cloud computing (with native support for Kubernetes, the Google Cloud Life Sciences API, Amazon AWS, TES ( https://www.ga4gh.org), and Microsoft Azure, the latter two in an upcoming release). 

Snakemake’s design ensures that scaling a workflow to a specific platform should only entail the modification of command line parameters. The workflow itself can remain untouched. Via configuration profiles, it is possible to persist and share the command line setup of Snakemake for any computing platform ( https://github.com/snakemake-profiles/doc). 

**Figure 4.**
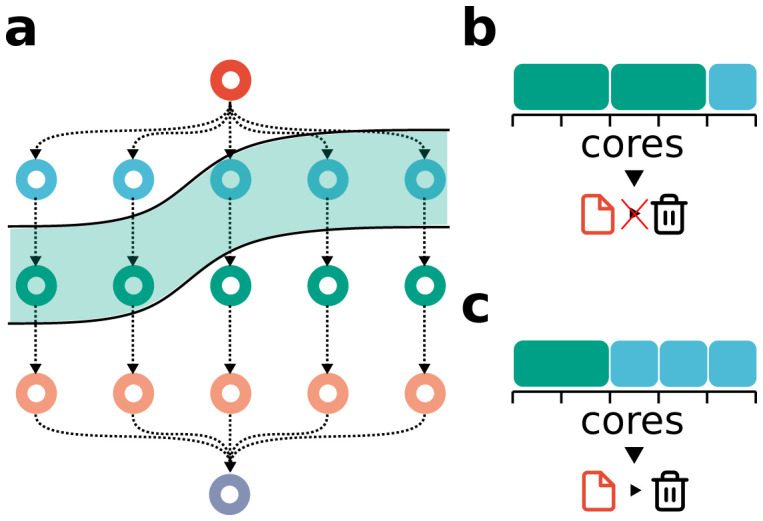
Snakemake scheduling problem. ( **a**) Example workflow DAG. The greenish area depicts the jobs that are ready for scheduling (because all input files are present) at a given time during the workflow execution. We assume that the red job at the root generates a temporary file, which may be deleted once all blue jobs are finished. ( **b**) Suboptimal scheduling solution: two green jobs are scheduled, such that only one blue job can be scheduled and the temporary file generated by the red job has to remain on disk until all blue jobs are finished in a subsequent scheduling step. ( **c**) Optimal scheduling solution: the three blue jobs are scheduled, such that the temporary file generated by the red job can be deleted afterwards.

***2.5.1 Job scheduling***. Because of their dependencies, not all jobs in a workflow can be executed at the same time. Instead, one can imagine partitioning the DAG of jobs into three sections: those that are already finished, those that have already been scheduled but are not finished yet, and those that have not yet been scheduled ( Figure 4a). Let us call the jobs in the latter partition *J ^o^*, the set of *open* jobs. Within *J ^o^*, all jobs that have only incoming edges from the partition of finished jobs (or no incoming edge at all) can be scheduled next. We call this the set *J* of *pending* jobs. The scheduling problem a workflow manager like Snakemake has to solve is to select the subset *E* ⊆ *J* that leads to an efficient execution of the workflow, while not exceeding the given resources like hard drive space, I/O capacity and CPU cores. Snakemake solves the scheduling problem at the beginning of the workflow execution and whenever a job has finished and new jobs become pending.

Efficiency of execution is evaluated according to three criteria. First, execution should be as fast as possible. Second, high-priority jobs should be preferred (Snakemake allows prioritization of jobs via the workflow definition and the command line). Third, temporary output files should be quickly deleted (Snakemake allows output files to be marked as temporary, which leads to their automatic deletion once all consuming jobs have been finished). An example is shown in Figure 4.

When running Snakemake in combination with cluster or cloud middleware (Slurm, PBS, LSF, Kubernetes, etc.), Snakemake does not need to govern available resources since that is handled by the middleware (hence, constraint ( 2) in Table 1 can be dropped). Instead, Snakemake can pass all information about job resource requirements (threads, memory, and disk) to the middleware, which can use this information to choose the best fitting machine for the job. Nevertheless, the number of jobs that shall be queued/running at a time is usually restricted in such systems, so that Snakemake still has to select a subset of jobs *E* ⊆ *J* as outlined above. In particular, minimizing the lifetime of temporary files and maximizing priority is still of high relevance, such that the scheduling problem still has to be solved, albeit without the resource constraints (resource requirements of selected jobs are simply passed to the middleware).

**Table 1.** Mixed integer linear program for Snakemake’s scheduling problem.

**Objective:** $\begin{array}{l} \;\text{Maximize}\;\text{2}U_{\text{c}}\cdot 2S\cdot \sum_{j\in J}p_{j}\cdot x_{j}+2S\cdot \sum_{j\in J}u_{\text{c},j}\cdot x_{j}\\ \;+S\cdot \sum_{f\in F}S_{f}\cdot \gamma_{f}+\sum_{f\in F}S_{f}\cdot \delta_{f}\;(1)\\ \;\text{subject}\;\text{to}\\ \;\begin{array}{c} \;x_{j}\in \{0,1\}\;\text{for}\;\text{all}\;j\in J,\\ \;\gamma_{f}\in \{0,1\}\;\;\text{for}\;\text{all}\;f\in F,\\ \;\delta_{f}\in [0,1]\;\text{for}\;\text{all}\;f\in F,\\ \;\sum_{j\in J}x_{j}\cdot u_{r,j}\leq \;U_{r}\;\text{for}\;\text{all}\;r\in R,\;(2) \end{array}\\ \begin{array}{c} \;\delta_{f}\leq \;\frac{\sum_{j\in J}\;x_{j}\cdot z_{f,j}}{\sum_{j\in J^{o}}\;z_{f,j}}\;\text{for}\;\text{all}\;f\in F,\;(3)\\ \;\gamma_{f}\leq \;\delta_{f}\;\text{for}\;\text{all}\;f\in F.\;(4) \end{array} \end{array}$	**Variables:** binary ( *x _j_*) _*j*∈ *J*_ : do we schedule job *j* ∈ *J*? binary ( *γ*_*f*_) _*f*∈ *F*_ : can we delete file *f* ∈ *F*? continuous ( *δ _f_*) _*f*∈ *F*_ ∈ [0, 1]: lifetime fraction of *f* ; see ( 3) **Parameters:** *p _j_* ∈ ℕ : priority of job *j* ∈ *J* *u _r,j_* ∈ ℕ : *j*’s usage of resource *r* *z _f,j_* : does job *j* ∈ *J ^o^* need file *f* ? *U _r_* ∈ ℕ : free capacity of resource *r* *S _f_* ∈ ℕ : size of file *f* *S* ∈ ℕ : sum of file sizes $\sum_{f}S_{f}$

We solve the scheduling problem via a mixed integer linear program (MILP) as follows. Let *R* be the set of resources used in the workflow (e.g., CPU cores and memory). By default, Snakemake only considers CPU cores which we indicate with c, i.e., *R* = {c}. Let *F* be the set of temporary files that are currently present. We first define constants for each pending job *j* ∈ *J*: Let *p _j_* ∈ ℕ be its priority, let *u _r,j_* ∈ ℕ be its usage of resource *r* ∈ *R*, and let *z _f,j_* ∈ {0, 1} indicate whether it needs temporary file *f* ∈ *F* as input ( *z _f,j_* = 1) or not ( *z _f,j_* = 0). Further, let *U _r_* be the free capacity of resource *r* ∈ *R* (initially what is provided to Snakemake on the command line; later what is left, given resources already used in running jobs). Let *S _f_* be the size of file *f* ∈ *F*, and let $S\;:\text{=}\sum_{f\in F}S_{f}$ be be total temporary file size (measured in some reasonable unit, such as MB). 

Next, we define indicator variables *x _j_* ∈ {0, 1}, for each job *j* ∈ *J*, indicating whether a job is selected for execution (1) or not (0). For each temporary file *f* ∈ *F*, we define a variable *δ _f_* ∈ [0, 1] indicating the fraction of consuming jobs that will be scheduled among all open jobs. We also call this variable the lifetime fraction of temporary file *f* . In other words, *δ _f_* = 1 means that all consuming jobs will be completed after this scheduling round has been processed, such that the lifetime of that file is over and it can be deleted. To indicate the latter, we further define a binary variable *γ _f_* ∈ {0, 1}, with *γ _f_* = 1 representing the case that *f* can indeed be deleted, in other words, *γ _f_* = 1 ⇔ *δ _f_* = 1.

Then, the scheduling problem can be written as the MILP depicted in Table 1. The maximization optimizes four criteria, represented by four separate terms in ( 1). First, we strive to prefer jobs with high priority. Second, we aim to maximize the number of used cores, i.e. the extent of parallelization. Third, we aim to delete existing temporary files as soon as possible. Fourth, we try to reduce the lifetime of temporary files that cannot be deleted in this pass.

We consider these four criteria in lexicographical order. In other words, priority is most important, only upon ties do we consider parallelization. Given ties while optimizing parallelization, we consider the ability to delete temporary files. And only given ties when considering the latter, we take the lifetime of all temporary files that cannot be deleted immediately into account. Technically, this order is enforced by multiplying each criterion sum with a value that is at least as high as the maximum value that the equation right of it can acquire. Unless the user explicitly requests otherwise, all jobs have the same priority, meaning that in general the optimization problem maximizes the number of used cores while trying to remove as many temporary files as possible. 

The constraints ( 2)–( 4) ensure that the variables have the intended meaning and that the computed schedule does not violate resource constraints. Constraint ( 2) ensures that the available amount *U _r_* of each resource *r* ∈ *R* is not exceeded by the selection. Constraint ( 3) (together with the fact that *δ _f_* is being maximized) ensures that *δ _f_* is ineed the lifetime fraction of temporary file *f* ∈ *F*. Note that the sum in the denominator extends over all open jobs, while the numerator only extends over pending jobs. Constraint ( 4) (together with the fact that *γ _f_* is being maximized) ensures that *γ _f_* = 0 if and only if *δ _f _* < 1 and hence calculates whether temporary file *f* ∈ *F* can be deleted.

Additional considerations and alternatives, which may be implemented in subsequent releases of Snakemake, are discussed in subsection 3.4. 

***2.5.2 Caching between workflows.*** While data analyses usually entail the handling of multiple datasets or samples that are specific to a particular project, they often also rely on retrieval and post-processing of common datasets. For example, in the life sciences, such datasets include reference genomes and corresponding annotations. Since such datasets potentially reoccur in many analyses conducted in a lab or at an institute, re-executing the analysis steps for retrieval and post-processing of common datasets as part of individual analyses would waste both disk space and computation time. 

Historically, the solution in practice was to compile shared resources with post-processed datasets that could be referred to from the workflow definition. For example, in the life sciences, this has led to the Illumina iGenomes resource ( https://support.illumina.com/sequencing/sequencing_software/igenome.html) and the GATK resource bundle ( https://gatk.broadinstitute.org/hc/en-us/articles/360035890811-Resource-bundle).

In addition, in order to provide a more flexible way of selection and retrieval for such shared resources, so-called “reference management” systems have been published, like Go Get Data ( https://gogetdata.github.io) and RefGenie ( http://refgenie.databio.org). Here, the logic for retrieval and post-processing is curated in a set of recipes or scripts, and the resulting resources can be automatically retrieved via command line utilities. The downside of all these approaches is that the transparency of the data analysis is hampered since the steps taken to obtain the used resources are hidden and less accessible for the reader of the data analysis.

**Figure 5.**
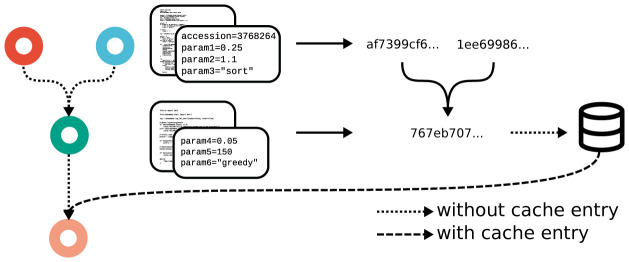
Blockchain-hashing based between workflow caching scheme of Snakemake. If a job is eligible for caching, its code, parameters, raw input files, software environment and the hashes of its dependencies are used to calculate a SHA-256 hash value, under which the output files are stored in a central cache. Subsequent runs of the same job (with the same dependencies) in other workflows can skip the execution and directly take the output files from the cache.

Snakemake provides a new, generic approach to the problem which does not have this downside (see Figure 5). Leveraging workflow-inherent information, Snakemake can calculate a hash value for each job that unambiguously captures exactly how an output file is generated, prior to actually generating the file. This hash can be used to store and lookup output files in a central cache (e.g., a folder on the same machine or in a remote storage). For any output file in a workflow, if the corresponding rule is marked as eligible for caching, Snakemake can obtain the file from the cache if it has been created before in a different workflow or by a different user on the same system, thereby saving computation time, as well as disk space (on local machines, the file can be linked instead of copied).

The hash value is calculated in the following way. Let *J* be the set of jobs of a workflow. For any job *j* ∈ *J*, let *c _j_* denote its code (shell command, script, wrapper, or notebook), let *P _j_* = {( *k _i_*, *v _i_*) | *i* = 0,..., *m*} be its set of parameters (with key *k _i_* and JSON-encoded value *v _i_*), let *F _j_* be its set of input files that are not created by any other job, and let *s _j_* be a string describing the associated software environment (either a container unique resource identifier, a Conda environment definition, or both). Then, assuming that job *j* ∈ *J* with dependencies *D _j_* ⊂ *J* is the job of interest, we can calculate the hash value as

$$\begin{array}{l} h(j)=h^{\prime }(\left(\overset{m}{\underset{i=0}{⊙}}k_{i}⊙v_{i}\right)⊙\;c_{j}⊙\\ \;\left(\underset{f\in F_{j}}{⊙}h^{\prime }(f)\right)⊙\;s_{j}⊙\\ \;\left(\underset{j^{\prime }\in D_{j}}{⊙}h(j^{\prime })\right)) \end{array}$$

with *h′* being the SHA-256 ^36^ hash function, ⊙ being the string concatenation, and ⊙ being the string concatenation of its operands in lexicographic order.

The hash function *h*( *j*) comprehensively describes everything that affects the content of the output files of job *j*, namely code, parameters, raw input files, the software environment and the input generated by jobs it depends on. For the latter, we recursively apply the hash function *h* again. In other words, for each dependency *j′* ∈ *D _j_* we include a hash value into the hash of job *j*, which is in fact the hashing principle behind blockchains used for cryptocurrency ^37^. The hash is only descriptive if the workflow developer ensures that the cached result is generated in a deterministic way. For example, downloading from a URL that yields data which may change over time should be avoided.

***2.5.3 Graph partitioning.*** A data analysis workflow can contain diverse compute jobs, some of which may be long-running, and some which may complete quickly. When executing a Snakemake workflow in a cluster or cloud setting, by default, every job will be submitted separately to the underlying queuing system. For short-running jobs, this can result in a considerable overhead, as jobs wait in a queue, and may also incur additional delays or cost when accessing files from remote storage or network file systems. To minimize such overhead, Snakemake offers the ability to partition the DAG of jobs into subgraphs that will be submitted together, as a single cluster or cloud job.

**Figure 6.**
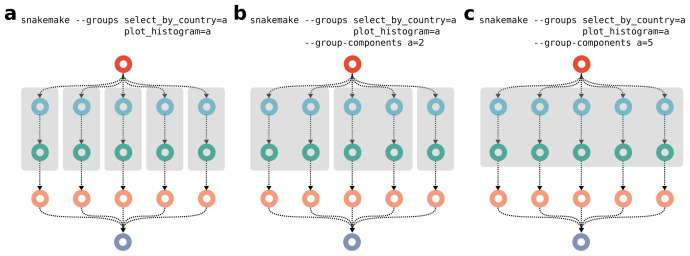
Job graph partitioning by assigning rules to groups. Two rules of the example workflow ( Figure 3a) are grouped together, ( **a**) spanning one connected component, ( **b**) spanning two connected components, and ( **c**) spanning five connected components. Resulting submitted group jobs are represented as grey boxes.

Partitioning happens by assigning rules to groups (see Figure 6). Upon execution, Snakemake determines connected subgraphs with the same assigned group for each job and submits such subgraphs together (as a so called *group job*) instead of submitting each job separately. For each group, it is in addition possible to define how many connected subgraphs shall be spanned when submitting (one by default). This way, it is possible to adjust the partition size to the needs of the available computational platform. The resource usage of a group job is determined by sorting involved jobs topologically, summing resource usage per level and taking the maximum over all levels.

***2.5.4 Streaming.*** Sometimes, intermediate results of a data analysis can be huge, but not important enough to store persistently on disk. Apart from the option to mark such files as temporary so that Snakemake will automatically delete them once no longer needed, it is also possible to instruct Snakemake to never store them on disk at all by directly streaming their content from the producing job to to the consuming job. This requires the producing and consuming jobs to run at the same time on the same computing node (then, the output of the producer can be written to a small in-memory buffer; on Unix, this is called a named pipe). Snakemake ensures this by submitting producer and consumer as a group job (see subsubsection 2.5.3).

## 3 Further considerations

### 3.1 Workflow composition

**Figure 7.**
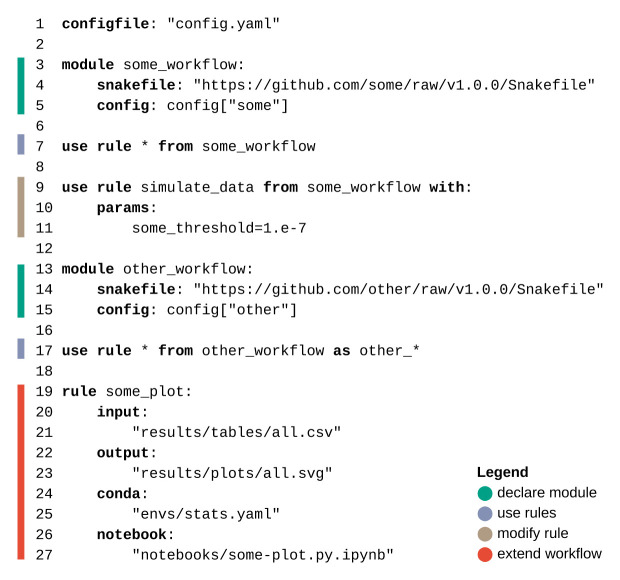
Workflow composition capabilities of Snakemake. Single or multiple external workflows can be declared as modules, along with the selection of all or specific rules. Properties of rules can be overwritten, and the analysis can be extended with further rules.

Upon development, data analyses are usually crafted with a particular aim in mind, for example being able to test a particular hypothesis, to find certain patterns in a given data type, etc. In particular with larger, long-running scientific projects, it can happen that data becomes increasingly multi-modal, encompassing multiple, orthogonal types that need completely different analyses. While a framework like Snakemake easily allows to develop a large integrative analyses over such diverse data types, such an analysis can also become very specific to a particular scientific project. When aiming for re-use of an analysis, it is often beneficial to keep it rather specific to some data type or not extend it beyond a common scope. Via the declaration of external workflows as modules, integration of such separately maintained workflows is well supported in Snakemake ( Figure 7). By referring to a local or remote Snakefile ( Figure 7, line 3–4) a workflow module can be declared, while configuration is passed as a Python dictionary object (line 5). The usage of all or specific rules from the workflow module can be declared (line 7), and properties of individual rules can be overwritten (e.g., params, input, output, line 9–11). This way, as many external workflows as needed can be composed into a new data analysis (line 13–17). Optionally, in order to avoid name clashes, rules can be renamed with the as keyword (line 17), analogously to the Python import mechanism. Moreover, the external workflows can be easily extended with further rules that generate additional output (line 19–27).

This way, both the plain use as well as extension and modification becomes immediately transparent from the source code. Often, data analyses extend beyond a template or production analysis that seemed appropriate at the beginning (at latest during the review of a publication). Hence, Snakemake’s workflow composition mechanism is also appropriate for the simple application of a published data analysis pipeline on new data. By declaring usage of the pipeline as a module as shown above, both the plain execution with custom configuration as well as an extension or modification becomes transparent. Moreover, when maintaining the applied analysis in a version controlled (e.g. git) repository, it does not need to host a copy of the source code of the original pipeline, just the customized configuration and any modifications.

### 3.2 Advanced workflow design patterns

**Figure 8.**
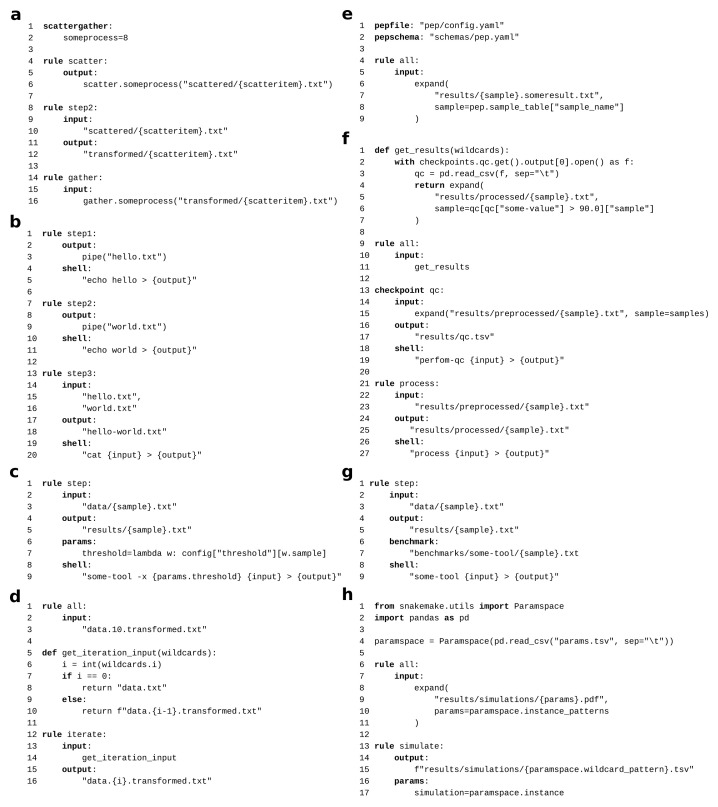
Additional design patterns for Snakemake workflows. For brevity only rule properties that are necessary to understand each example are shown (e.g. omitting log directives and shell commands or script directives). ( **a**) scatter/gather process, ( **b**) streaming, ( **c**) non-file parameters, ( **d**) iteration, ( **e**) sample sheet based configuration, ( **f**) conditional execution, ( **g**) benchmarking, ( **h**) parameter space exploration. See subsection 3.2 for details.

Figure 8 shows advanced design patterns which are less common but useful in certain situations. For brevity, only rule properties that are necessary to understand each example are shown (e.g. omitting log directives and shell commands or script directives). Below, we explain each example in detail.

**Scatter/gather processes ( Figure 8a)**. Snakemake’s ability to employ arbitrary Python code for defining a rule’s input and output files already enables any kind of scattering, gathering, and aggregations in workflows. Nevertheless, it can be more readable and scalable to use Snakemake’s explicit support for scatter/gather processes. A Snakemake workflow can have any number of such processes, each of which has a name (here someprocess). In this example, the rule scatter (line 4) splits some data into *n* items; the rule step2 (line 8) is applied to each item; the rule gather (line 14) aggregates over the outputs of step2 for each item. Thereby, *n* is defined via the scattergather directive (line 1) at the beginning, which sets *n* for each scatter/gather process in the workflow. In addition, *n* can be set via the command line via the flag --set-scatter. For example, here, we could set the number of scatter items to 16 by specifying --set-scatter someprocess=16. This enables the user to better scale the data analysis workflow to its computing platform, beyond the defaults provided by the workflow designer.

**Streaming ( Figure 8b)**. Snakemake allows to stream output between jobs, instead of writing it to disk (see subsubsection 2.5.4). Here, the output of rule step1 (line 1) and step2 (line 7) is streamed into rule step3 (line 13).

**Non-file parameters ( Figure 8c)**. Data analysis steps can need additional non-file input in the form of parameters, that are for example obtained from the workflow configuration (see subsection 2.1). Both input files and such non-file parameters can optionally be defined via a Python function, which is evaluated for each job, when wildcard values are known. In this example, we define a lambda expression (an anonymous function in Python), that retrieves a threshold depending on the value of the wildcard sample ( w.sample, line 7). Wildcard values are passed as the first positional argument to such functions (here w, line 7).

**Iteration ( Figure 8d).** Sometimes, a certain step in a data analysis workflow needs to be applied iteratively. Snakemake allows to model defining by setting the iteration count variable as a wildcard (here {i}, line 16). Then, an input function can be used to either request the output of the previous iteration (if i > 0, line 10) or the initial data (if i == 0, line 8). Finally, in the rule that requests the final iteration result, the wildcard {i} is set to the desired count (here 10, line 3).

**Sample sheet based configuration ( Figure 8e)**. Often, scientific experiments entail multiple samples, for which meta-information is known (e.g. gender, tissue etc. in biomedicine). Portable encapsulated projects (PEPs, https://pep.databio.org) are an approach to standardize such information and provide them in a shareable format. Snakemake workflows can be directly integrated with PEPs, thereby allowing to configure them via meta-information that is contained in the sample sheets defined by the PEP. Here, a pepfile (line 1) along with a validation schema (line 2) is defined, followed by an aggregation over all samples defined in the contained sample sheet.

**Conditional execution ( Figure 8f)**. By default, Snakemake determines the entire DAG of jobs upfront, before the first job is executed. However, sometimes the analysis path that shall be taken depends on some intermediate results. For example, this is the case when filtering samples based on quality control criteria. At the beginning of the data analysis, some quality control (QC) step is performed, which yields QC values for each sample. The actual analysis that shall happen afterwards might be only suitable for samples that pass the QC. Hence, one might have to filter out samples that do not pass the QC. Since the QC is an intermediate result of the same data analysis, it can be necessary to determine the part of the DAG that comes downstream of the QC only after QC has been finalized. Of course, one option is to separate QC and the actual analysis into two workflows, or defining a separate target rule for QC, such that it can be manually completed upfront, before the actual analysis is started. Alternatively, if QC shall happen automatically as part of the whole workflow, one can make use of Snakemake’s conditional execution capabilities. In the example, we define that the qc rule shall be a so-called checkpoint. Rules can depend on such checkpoints by obtaining their output from a global checkpoints object (line 2), that is accessed inside of a function, which is passed to the input directive of the rule (line 11). This function is re-evaluated after the checkpoint has been executed (and its output files are present), thereby allowing to inspect the content of the checkoint’s output files, and decide about the input files based on that. In this example, the checkpoint rule qc creates a TSV file, which the function loads, in order to extract only those samples for which the column "some-value" contains a value greater than 90 (line 6). Only for those samples, the file "results/processed/{sample}.txt" is requested, which is then generated by applying the rule process for each of these samples.

**Benchmarking ( Figure 8g**). Sometimes, a data analysis entails the benchmarking of certain tools in terms of runtime, CPU, and memory consumption. Snakemake directly supports such benchmarking by defining a benchmark directive in a rule (line 7). This directive takes a path to a TSV file. Upon execution of a job spawned from such a rule, Snakemake will constantly measure CPU and memory consumption, and store averaged results together with runtime information into the given TSV file. Benchmark files can be input to other rules, for example in order to generate plots or summary statistics.

**Parameter space exploration ( Figure 8h)**. In Python (and therefore also with Snakemake), large parameter spaces can be represented very well via Pandas ^38, 39^ data frames. When such a parameter space shall be explored by the application of a set of rules to each instance of the space (i.e., each row of the data frame), the idiomatic approach in Snakemake is to encode each data frame column as a wildcard and request all occuring combinations of values (i.e., the data frame rows), by some consuming rule. However, with large parameter spaces that have a lot of columns, the wildcard expressions could become cumbersome to write down explicitly in the Snakefile. Therefore, Snakemake provides a helper called Paramspace, which can wrap a Pandas data frame (this functionality was inspired by the JUDI workflow management system ^16^ https://pyjudi.readthedocs.io). The helper allows to retrieve a wildcard pattern (via the property wildcard_pattern) that encodes each column of the data frame in the form name~{name} (i.e., column name followed by the wildcard/wildcard value). The wildcard pattern can be formatted into input or output file names of rules (line 15). The method instance of the Paramspace object, automatically returns the corresponding data frame row (as a Python dict) for given wildcard values (here, that method is automatically evaluated by Snakemake for each instance of the rule simulate, line 17). Finally, aggregation over a parameter space becomes possible via the property instance_patterns, which retrieves a concrete pattern of above form for each data frame row. Using the expand helper, these patterns can be formatted into a file path (line 8–11), thereby modelling an aggregation over the entire parameter space. Naturally, filtering rows or columns on the paramspace via the usual Pandas methods allows to generate sub-spaces. 

### 3.3 Readability

Statements in Snakemake workflow definitions fall into seven categories:

1. a natural language word, followed by a colon (e.g. input: and output:),
2. the word “rule”, followed by a name and a colon (e.g. rule convert_to_pdf:),
3. a quoted filename pattern (e.g. "{prefix}.pdf"),
4. a quoted shell command,
5. a quoted wrapper identifier,
6. a quoted container URL
7. a Python statement.

Below, we list the rationale of our assessment for each category in Figure 3:

1. The natural language word is either trivially understandable (e.g. input: and output:) or understandable with technical knowledge ( container: or conda:). The colon straightforwardly shows that the content follows next. Only for the wrapper directive ( wrapper:) one needs to have the Snakemake specific knowledge that it is possible to refer to publicly available tool wrappers.
2. The word “rule” is trivially understandable, and when carefully choosing rule names, at most domain knowledge is needed for understanding such statements.
3. Filename patterns can mostly be understood with domain knowledge, since the file extensions should tell the expert what kind of content will be used or created. We hypothesize that wildcard definitions (e.g. {country}) are straightforwardly understandable as a placeholder.
4. Shell commands will usually need domain and technical knowledge for understanding.
5. Wrapper identifiers can be understood with Snakemake knowledge only, since one needs to know about the central tool wrapper repository of Snakemake. Nevertheless, with only domain knowledge one can at least conclude that the mentioned tool (last part of the wrapper ID) will be used in the wrapper.
6. A container URL will usually be understandable with technical knowledge.
7. Python statements will either need technical knowledge or Snakemake knowledge (when using the Snakemake API, as it happens here with the expand command, which allows to aggregate over a combination of wildcard values).

### 3.4 Scheduling

While the first releases of Snakemake used a greedy scheduler, the current implementation aims at using more efficient schedules by solving a mixed integer linear program (MILP) whenever there are free resources. The current implementation already works well; still, future releases may consider additional objectives:

- The current formulation leads to fast removal of existing temporary files. In addition, one may control creation of temporary files in the first place, such that only limited space is occupied by temporary files at any time point during workflow execution.
- It may also be beneficial to initially identify bottleneck jobs in the graph and prioritize them automatically instead of relying on the workflow author to prioritize them.

Because we consider different objectives hierarchically and use large constants in the objective function, currently a high solver precision is needed. If more objectives are considered in the future, an alternative hierarchical formulation may be used: First find the optimal objective value for the first (or the first two) objectives; then solve another MILP that maximizes less important objectives and ensures via constraints that the optimality of the most important objective(s) is not violated, or stays within, say, 5% of the optimal value.

We also need to mention a technical detail about the interaction between the scheduler and streams ( subsection 3.2). Some jobs that take part in handling a data stream may effectively use zero cores (because they mostly wait for data and then only read or write data), i.e. they have *u*_c, *j*_ = 0 in the MILP notation, which means that they do not contribute to the objective function. We thus replace the MILP objective term that maximizes paralellization $(\sum_{j\in J}\;u_{\text{c},j}\cdot x_{j})$ by the modified term $\sum_{j\in J}\;\max\{u_{\text{c},j},\;1\}\cdot x_{j}$ to ensure that the weight of any *x _j_* within the sum is at least 1.

### 3.5 Performance

**Figure 9.**
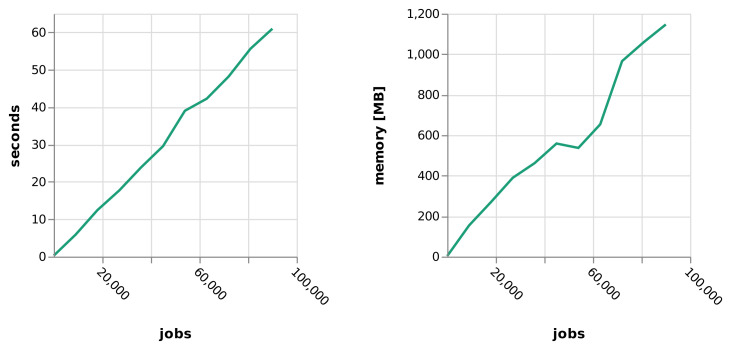
Runtime and memory usage of Snakemake while building the graph of jobs depending on the number of jobs in the workflow. The Snakemake workflow generating the results along with a self-contained Snakemake report that connects results and provenance information is available at https://doi.org/10.5281/zenodo.4244143.

When executing a data analysis workflow, running time and resource usage is dominated by the executed jobs and the performance of the libraries and tools used in these. Nevertheless, Snakemake has to process dependencies between jobs, which can incur some startup time until the actual workflow is executed. In order to provide an estimate on the amount of time and memory needed for this computation, we took the example workflow from Figure 3 in the main manuscript and artificially inflated it by replicating the countries in the input dataset. By this, we generated workflows of 10 to 90,000 jobs. Then, we benchmarked runtime and memory usage of Snakemake for computing the entire graph of jobs on these on a single core of an Intel Core i5 CPU with 1.6 GHz, 8 GB RAM and a Lenovo PCIe SSD (LENSE20512GMSP34MEAT2TA) ( Figure 9). It can be seen that both runtime and memory increase linearly, starting from 0.2 seconds with 2.88 MB for 11 jobs and reaching 60 seconds with 1.1 GB for 90,000 jobs.

For future releases of Snakemake, we plan to further improve performance, for example by making use of PyPy ( https://www.pypy.org), and by caching dependency resolution results between subsequent invocations of Snakemake. 

## 4 Conclusion

While having been almost the holy grail of data analysis workflow management in recent years and being certainly of high importance, reproducibility alone is not enough to sustain the hours of work that scientists invest in crafting data analyses. Here, we outlined how the interplay of automation, scalability, portability, readability, traceability, and documentation can help to reach beyond reproducibility, making data analyses adaptable and transparent. Adaptable data analyses can not only be repeated on the same data, but also be modified and extended for new questions or scenarios, thereby greatly increasing their value for both the scientific community and the original authors. While reproducibility is a necessary property for checking the validity of scientific results, it is not sufficient. Being able to reproduce exactly the same figure on a different machine tells us that the analysis is robust and valid from a technical perspective. However, it does not tell anything about the methodological validity (correctness of statistical assumptions, avoidance of overfitting, etc.). The latter can only be secured by having a transparent yet accessible view on the analysis code.

By analyzing its readability and presenting its modularization, portability, reporting, scheduling, caching, partitioning, and streaming abilities, we have shown how Snakemake supports all these aspects, thereby providing a comprehensive framework for sustainable data analysis, and enabling an ergonomic, unified, combined representation of any kind of analysis step, from raw data processing, to quality control and fine-grained, interactive exploration and plotting of final results.

## Software availability

Snakemake is available as MIT licensed open source software (homepage: https://snakemake.github.io, repository: https://github.com/snakemake/snakemake) and can be installed via Bioconda ^40^. 

## Data availability 

The Snakemake workflow generating the results presented in this work, along with the corresponding Snakemake report connecting results and provenance information is available at https://doi.org/10.5281/zenodo.4244143 ^28^.

Data are available under the terms of the Creative Commons Attribution 4.0 International license (CC-BY 4.0).

## Author information

Felix Mölder has designed and implemented the job scheduling mechanism ( subsubsection 2.5.1; supervised by Johannes Köster and Sven Rahmann) and edited the manuscript. Kim Philip Jablonski has designed and implemented Jupyter notebook integration ( subsubsection 2.2.1) and edited the manuscript. Michael Hall and Brice Letcher have designed and implemented automated code formatting ( subsubsection 2.2.2) and Brice Letcher has edited the manuscript. Vanessa Sochat has designed and implemented the Google Cloud Life Sciences API execution backend, as well as various improvements to Google storage support ( subsection 2.5) and edited the manuscript. Soohyun Lee has designed and implemented the AWS execution backend via integration with Tibanna ( subsection 2.5). Sven O. Twardziok and Alexander Kanitz have designed and implemented the TES execution backend ( subsection 2.5). Andreas Wilm has designed and implemented the Microsoft Azure execution backend ( subsection 2.5) and edited the manuscript. Manuel Holtgrewe has designed and implemented benchmarking support ( subsection 3.2). Jan Forster has designed and implemented meta-wrapper support ( subsubsection 2.2.1). Christopher Tomkins-Tinch has designed and implemented remote storage support ( subsection 2.1) and edited the manuscript. Sven Rahmann has edited the manuscript. Sven Nahnsen has provided the initial idea of using blockchain hashing to fingerprint output files a priori ( subsubsection 2.5.2). Johannes Köster has written the manuscript and implemented all other features that occur in the text but are not explicitly mentioned in above listing. All authors have read and approved the manuscript.
