# Supplementary material for: Sustainable data analysis with Snakemake
Source: F1000Res. 2025 Sep 23;10:33. Originally published 2021 Jan 18. [Version 3] doi: 10.12688/f1000research.29032.3 (PMC8114187; doi:10.12688/f1000research.29032.3)
Supplement: Peer review history

# Reviewer report: Reviewer response for version 3

**DOI:** 10.5256/f1000research.173023.r416658

**Published:** 2025-10-10

**Authors:** Caroline C. Friedel

**Affiliations:** 1 Institute of Informatics, LMU Munich, Munich, Germany

I have no further comments to make.

Is the rationale for developing the new method (or application) clearly explained?

Yes

Is the description of the method technically sound?

Yes

Are the conclusions about the method and its performance adequately supported by the findings presented in the article?

Partly

If any results are presented, are all the source data underlying the results available to ensure full reproducibility?

Yes

Are sufficient details provided to allow replication of the method development and its use by others?

Yes

Reviewer Expertise:

Bioinformatics

I confirm that I have read this submission and believe that I have an appropriate level of expertise to confirm that it is of an acceptable scientific standard.

# Reviewer report: Reviewer response for version 2

**DOI:** 10.5256/f1000research.56004.r83481

**Published:** 2021-05-11

**Authors:** Caroline C. Friedel

**Affiliations:** 1 Institute of Informatics, LMU Munich, Munich, Germany

**Response 2**

*"Indeed, we agree that our initial example was lacking parameter definitions via the params directive, which are indeed quite ubiquitous in practice. We have extended our example accordingly."*

 While the params directive is now included in the new example in Figure 7, it is still mentioned as one of the design patterns that are less common in section 3.2. I would suggest reordering the design patterns in 3.2 to first mention the params directive and then the others and change the wording "...advanced design patterns which are less common but useful in certain situations" to "...advanced design patterns. Some of these are less common but useful in certain situations."

 My other comments were satisfactorily addressed.

Is the rationale for developing the new method (or application) clearly explained?

Yes

Is the description of the method technically sound?

Yes

Are the conclusions about the method and its performance adequately supported by the findings presented in the article?

Partly

If any results are presented, are all the source data underlying the results available to ensure full reproducibility?

Yes

Are sufficient details provided to allow replication of the method development and its use by others?

Yes

Reviewer Expertise:

Bioinformatics

I confirm that I have read this submission and believe that I have an appropriate level of expertise to confirm that it is of an acceptable scientific standard.

# Reviewer report: Reviewer response for version 1

**DOI:** 10.5256/f1000research.32078.r79773

**Published:** 2021-03-11

**Authors:** Caroline C. Friedel

**Affiliations:** 1 Institute of Informatics, LMU Munich, Munich, Germany

The authors present an updated version of their workflow management system snakemake. Here, the authors focus on particular aspects important for sustainable data analysis, in particular automation, readability, portability, documentation and scalability. This is followed by a section on "further consideration", which presents specific workflow patterns, more details on their readability analysis, some more details on scheduling as well as a performance analysis. Apart from the performance analysis at the end, these "further considerations" aspects would be more appropriate earlier in the manuscript. In particular, the scheduling considerations (3.3) would be more appropriate in the section on scalability (2.5), where scheduling is extensively described. The readability considerations (3.2) would be more appropriate in the readability section earlier (2.2), in particular as the latter refers extensively to these considerations. The current structure ironically reduces readability of the article.

 The advanced workflow design patterns are more difficult to place, but would be more appropriate somewhere before readability as they are not quite as easy to read as the simple example at the beginning and their readability should be discussed. While the authors state that they are "less common but useful in certain situations", I would argue that some of them, in particular "Non-file parameters" (Fig. 7c) should be commonly used. Most bioinformatics tools one would want to use in a workflow, have multiple non-file parameters where one would not necessarily use the default values (if there even are defaults).

 Apart from these issues, the authors present their case well on what they consider to be important for sustainable data analysis and show that snakemake is both well maintained, and commonly used, not only in the bioinformatics community. The section on job scheduling is very extensive, but I am also missing some details on how snakemake interacts with cluster scheduling software like SLURM etc. The question remains whether that complex mixed integer linear program for scheduling is still relevant or necessary if jobs will be submitted to a cluster with an own load-balancing software anyway. Another point that should be addressed is how or if different computing environments could be combined, e.g. if one has both a high-memory machine available for memory-intensive jobs and a separate computing cluster. Would one have to either run all jobs in the same environment or separate the workflow into two workflows that are run separately?

 The main issue I have with the manuscript is that the authors overstate the readability of snakemake workflows. Readability is extensively discussed on a very simply workflow even with a sort of quantification of the readability of the workflow. However, this workflow would also be pretty easy to read as a simple bash script, so I do not think this is an appropriate example to show how readable snakemake workflows are. Furthermore, several of the lines they consider "trivial", in my opinion, still require some understanding of snakemake, make or programming. I have previously worked with snakemake, though not recently, and in my experience the rule structure is not easy to mentally parse if one is not familiar with it. Moreover, even if every single line were trivial the whole workflow could still be difficult to understand due to the dependencies which are implicitly created through use of common in- or output files. This can very easily lead to a very complex structure, in particular since the order in which rules are given does not have to be in order of their dependencies. Their argument is also somewhat contradicted by the advanced workflow design patterns presented later, which are not that easy to read even with the explanation.

 I think the article would benefit if instead of trying to quantify the readability of the simple workflow, the authors would focus more on the approaches they included in snakemake for improving readability of workflows, i.e. modularization and standardized code linting and formatting. In particular, I would be interested in hearing more details on the snakefmt tool and the recommended best practices.

Is the rationale for developing the new method (or application) clearly explained?

Yes

Is the description of the method technically sound?

Yes

Are the conclusions about the method and its performance adequately supported by the findings presented in the article?

Partly

If any results are presented, are all the source data underlying the results available to ensure full reproducibility?

Yes

Are sufficient details provided to allow replication of the method development and its use by others?

Yes

Reviewer Expertise:

Bioinformatics

I confirm that I have read this submission and believe that I have an appropriate level of expertise to confirm that it is of an acceptable scientific standard, however I have significant reservations, as outlined above.

# Response

**Published:** 2021-04-08

**Authors:** Johannes Köster

**Affiliations:** University of Duisburg-Essen, Germany

Thanks a lot for the comprehensive assessment of the manuscript. This was very helpful and we are confident that your suggestions have significantly improved the article. Answers to individual comments can be found below.

**Comment 1**

 Here, the authors focus on particular aspects important for sustainable data analysis, in particular automation, readability, portability, documentation and scalability. This is followed by a section on "further consideration", which presents specific workflow patterns, more details on their readability analysis, some more details on scheduling as well as a performance analysis. Apart from the performance analysis at the end, these "further considerations" aspects would be more appropriate earlier in the manuscript. In particular, the scheduling considerations (3.3) would be more appropriate in the section on scalability (2.5), where scheduling is extensively described. The readability considerations (3.2) would be more appropriate in the readability section earlier (2.2), in particular as the latter refers extensively to these considerations. The current structure ironically reduces readability of the article.

**Response 1**

 Thanks a lot for this suggestion. We had indeed moved around these sections several times before submission. In the end, we thought the current structure is best at addressing a potentially diverse readership (technical and non-technical, seeking for a quick overview or for in-depth details, experienced users and beginners) with section 2 providing a comprehensive overview and section 3 showing additional details for particularly interested readers.

 We have added an explanation of the concept to the end of the introduction, and hope that this clarifies the intention and hopefully diminishes the negative effects of the split.

**Comment 2**

 The advanced workflow design patterns are more difficult to place, but would be more appropriate somewhere before readability as they are not quite as easy to read as the simple example at the beginning and their readability should be discussed. While the authors state that they are "less common but useful in certain situations", I would argue that some of them, in particular "Non-file parameters" (Fig. 7c) should be commonly used. Most bioinformatics tools one would want to use in a workflow, have multiple non-file parameters where one would not necessarily use the default values (if there even are defaults).

**Response 2**

 Thanks a lot for these important thoughts. Indeed, we agree that our initial example was lacking parameter definitions via the params directive, which are indeed quite ubiquitous in practice. We have extended our example accordingly.

**Comment 3**

 Apart from these issues, the authors present their case well on what they consider to be important for sustainable data analysis and show that snakemake is both well maintained, and commonly used, not only in the bioinformatics community. The section on job scheduling is very extensive, but I am also missing some details on how snakemake interacts with cluster scheduling software like SLURM etc. The question remains whether that complex mixed integer linear program for scheduling is still relevant or necessary if jobs will be submitted to a cluster with an own load-balancing software anyway. 

**Response 3**

 Indeed, the scheduling problem description was lacking an explanation about what happens in a cluster/cloud setting. We have extended the text accordingly. In brief: resource requirements are passed to the cluster/cloud middleware, but the scheduling problem still has to be solved in order to prioritze jobs and minimize the lifetime of temporary files. It becomes a bit less constrained though.

**Comment 4**

 Another point that should be addressed is how or if different computing environments could be combined, e.g. if one has both a high-memory machine available for memory-intensive jobs and a separate computing cluster. Would one have to either run all jobs in the same environment or separate the workflow into two workflows that are run separately?

**Response 4**

 This is indeed a very good point. For addressing execution on multiple machines, Snakemake entirely relies on cluster or cloud middleware. By specifying resource requirements per rule (or dynamically per job), which are passed to the middleware, it is of course possible to run different jobs on different types of machines. What is currently not possible is to combine different execution backends like two different cluster systems or a cluster and a local high memory machine. We have updated the text accordingly.

**Comment 5**

 The main issue I have with the manuscript is that the authors overstate the readability of snakemake workflows. Readability is extensively discussed on a very simply workflow even with a sort of quantification of the readability of the workflow. However, this workflow would also be pretty easy to read as a simple bash script, so I do not think this is an appropriate example to show how readable snakemake workflows are. Furthermore, several of the lines they consider "trivial", in my opinion, still require some understanding of snakemake, make or programming. I have previously worked with snakemake, though not recently, and in my experience the rule structure is not easy to mentally parse if one is not familiar with it. Moreover, even if every single line were trivial the whole workflow could still be difficult to understand due to the dependencies which are implicitly created through use of common in- or output files. This can very easily lead to a very complex structure, in particular since the order in which rules are given does not have to be in order of their dependencies. Their argument is also somewhat contradicted by the advanced workflow design patterns presented later, which are not that easy to read even with the explanation.

 I think the article would benefit if instead of trying to quantify the readability of the simple workflow, the authors would focus more on the approaches they included in snakemake for improving readability of workflows, i.e. modularization and standardized code linting and formatting. In particular, I would be interested in hearing more details on the snakefmt tool and the recommended best practices.

**Response 5**

 This is indeed a valid point, thanks a lot for bringing it up. We have rewritten the readability section to better reflect that the presented example shows an ideal, quite simple situation, that might be impossible to reach for parts of workflows in practice (but nevertheless should be aimed for). We have added advice on how to use modularization to help the reader of a workflow in such cases.

 We still think that the example is appropriate (in combination with the mentioned changes in the text). 

 While it is indeed simple, a bash script that would contain all the work that Snakemake is doing behind the scenes (checking file consistency, scheduling, various execution backends, parallelization, software stack deployment, etc.) would be much longer and less readable.

 We have also checked the triviality claims in all lines again (the numbers did change slightly). Of course, we'd be grateful to discuss or directly modify specific examples where you might still disagree with our judgement after the fixes.

 We agree that the dependency structure can be sometimes complicated and we have therefore added some sentences that clarify this. Here, it is important to mention Snakemake's ability to visualize dependencies and to automatically generate interactive reports. Since the latter are of particular value for peeking into the codebase without needing to understand the entire workflow, we have further extended the corresponding section (2.4).

 We are thankful for the suggestion to elaborate on other measures for improving readability and have therefore extended our section on the linter and formatter (Section 2.2.2). Further, we have added some additional sentences about modularization and how to use it best for ensuring readability (Section 2.2.1).

# Reviewer report: Reviewer response for version 1

**DOI:** 10.5256/f1000research.32078.r77869

**Published:** 2021-02-15

**Authors:** Michael Reich

**Affiliations:** 1 Department of Medicine, University of California, San Diego, San Diego, CA, USA

The authors describe Snakemake, a text-based pipeline execution system that builds on the principles of the Unix 'make' utility to include optimized job scheduling, extensive specification of compute resources, advanced workflow features, integration with package managers and container technologies, and result caching. The authors identify where Snakemake exists within the large ecosystem of pipeline management systems and describe the motivating principles of Snakemake in terms of a hierarchy of sustainable data analysis that includes reproducibility, accessibility, transparency, and other objectives. They then walk through several examples illustrating the scope and use of the system. This paper updates and extends the original Snakemake publication of Köster *et al*., Bioinformatics 2012 ^1^. The value of Snakemake to the field of bioinformatics is well-established, and the paper provides usage statistics and citations to reinforce this point.

 The paper thoroughly describes the features of Snakemake and the necessary background to understand their use. While it is possible to understand a Snakemake workflow at a high level with little programming knowledge, experience with Unix, Python, and an understanding of how 'make' works are necessary to author Snakemake workflows. The authors balance the conceptual overview with well-chosen usage examples that are simple enough to understand and make clear how the example can generalize to other cases. They also describe in detail the job scheduling algorithm that snakemake uses. The paper would benefit from a brief discussion of how snakemake interacts with load-balancing software (such as SLURM, Torque, LSF, etc.), because of the focus on the details of executing jobs. This information is in the documentation on the Snakemake web site, but a conceptual overview in the paper would help the reader to understand this relationship.

 Information necessary to install and use snakemake is available on the snakemake web site as referenced in the paper. The instructions and tutorial are comprehensive and understandable, and the progressive exercises give a good feel for the snakemake paradigm. Given the ease and popularity of container technology, it would be useful to include how to run snakemake from a Docker container in the tutorial. I was able to do this easily from the official snakemake container on Dockerhub and think it would be a helpful alternative in the documentation to installing Miniconda.

 In general, this paper provides a comprehensive introduction and overview of Snakemake and serves as an effective entry point to this popular system.

Is the rationale for developing the new method (or application) clearly explained?

Yes

Is the description of the method technically sound?

Yes

Are the conclusions about the method and its performance adequately supported by the findings presented in the article?

Yes

If any results are presented, are all the source data underlying the results available to ensure full reproducibility?

No source data required

Are sufficient details provided to allow replication of the method development and its use by others?

Yes

Reviewer Expertise:

Bioinformatics; reproducible research; workflow systems; open science

I confirm that I have read this submission and believe that I have an appropriate level of expertise to confirm that it is of an acceptable scientific standard.

## References

[1] : Snakemake--a scalable bioinformatics workflow engine. *Bioinformatics* .2012;28(19) : 10.1093/bioinformatics/bts480 2520-2 10.1093/bioinformatics/bts480 22908215

# Response

**Published:** 2021-04-08

**Authors:** Johannes Köster

**Affiliations:** University of Duisburg-Essen, Germany

Thanks a lot for the assessment of our manuscript. Your comments have led to several important improvements. Answers to individual comments can be found below.

**Comment 1**

 The paper would benefit from a brief discussion of how snakemake interacts with load-balancing software (such as SLURM, Torque, LSF, etc.), because of the focus on the details of executing jobs. This information is in the documentation on the Snakemake web site, but a conceptual overview in the paper would help the reader to understand this relationship.

**Response 1**

 Indeed, the paper failed to explicitly mention how this is handled. We have now added an explaining paragraph to section 2.5.1. In brief: resource requirements are passed to the middleware, but the scheduling problem still has to be solved in order to prioritze jobs and minimize the lifetime of temporary files. It becomes a bit less constrained though.

**Comment 2**

 Information necessary to install and use snakemake is available on the snakemake web site as referenced in the paper. The instructions and tutorial are comprehensive and understandable, and the progressive exercises give a good feel for the snakemake paradigm. Given the ease and popularity of container technology, it would be useful to include how to run snakemake from a Docker container in the tutorial. I was able to do this easily from the official snakemake container on Dockerhub and think it would be a helpful alternative in the documentation to installing Miniconda.

**Response 2**

 Offering further ways of performing the tutorial is indeed a good idea. We have considered providing instructions for performing the tutorial from a container, but felt that this would complicate editing and interaction too much. However, we now instead provide a Gitpod setup for the tutorial, which allows to directly run it, without any setup needed, from inside a browser window which offers both an editor and a terminal based on the Eclipse Theia IDE (see here).
